# Supplementary material for: A comparative study of machine learning and deep learning algorithms to classify cancer types based on microarray gene expression data
Source: PeerJ Comput Sci. 2020 Apr 13;6:e270. doi: 10.7717/peerj-cs.270 (PMC7924492; doi:10.7717/peerj-cs.270)
Supplement: Supplemental Information 1 — These commands were executed in Rstudio to calculate if the accuracies of the ML algorithms are statistically different. Also, these commands were used to find out if the tuning process produced significant changes in the accuracies at a confidence level of 95%. [file peerj-cs-06-270-s001.pdf]

Supplementary Material 1. R commands for performing significance tests

```
######## significance tests of non-tunned algorithms vs tunned
algorithms

## KNN
x = c(78.3, 82.03)
n = c(100, 100)
prop.test(x,n,alternative = "two.sided", conf.level = 0.95)
# 95 percent confidence interval:
# -0.15770686  0.08310686

## SVC
x = c(10.82, 81.98)
n = c(100, 100)
prop.test(x,n,alternative = "two.sided", conf.level = 0.95)
# 95 percent confidence interval:
# -0.8184589 -0.6047411

## Logistic Regression
x = c(90.6, 90.6)
n = c(100, 100)
prop.test(x,n,alternative = "two.sided", conf.level = 0.95)
# 95 percent confidence interval:
# -0.08088931  0.08088931

## Multi-Layer Perceptron
x = c(79.89, 83.40)
n = c(100, 100)
prop.test(x,n,alternative = "two.sided", conf.level = 0.95)
```

```
# 95 percent confidence interval:
# -0.152291  0.082091

## Kmeans
x = c(10.16, 68.34)
n = c(100, 100)
prop.test(x,n,alternative = "two.sided", conf.level = 0.95)
# 95 percent confidence interval:
# -0.7005103 -0.4630897

## Random Forest
x = c(66.75, 72.69)
n = c(100, 100)
prop.test(x,n,alternative = "two.sided", conf.level = 0.95)
# 95 percent confidence interval:
# -0.19648974  0.07768974

## Decision Tree
x = c(69.78, 66.04)
n = c(100, 100)
prop.test(x,n,alternative = "two.sided", conf.level = 0.95)
# 95 percent confidence interval:
# -0.1018903  0.1766903

####### significance test of best algorithms (LR and CNN)
#     LR      CNN
x = c(90.6, 94.43)
n = c(100, 100)
prop.test(x,n,alternative = "two.sided", conf.level = 0.95)
# 95 percent confidence interval:
```

-0.12104649 0.04444649
