# Supplementary material for: A comparative study of machine learning and deep learning algorithms to classify cancer types based on microarray gene expression data
Source: PeerJ Comput Sci. 2020 Apr 13;6:e270. doi: 10.7717/peerj-cs.270 (PMC7924492; doi:10.7717/peerj-cs.270)
Supplement: Supplemental Information 2 — These hierarchical maps were generated by data without transformation and deleting their labels. Clustering approaches demonstrate whether the data contain relevant patterns for grouping. [file peerj-cs-06-270-s002.pdf]

Supplementary Material 2

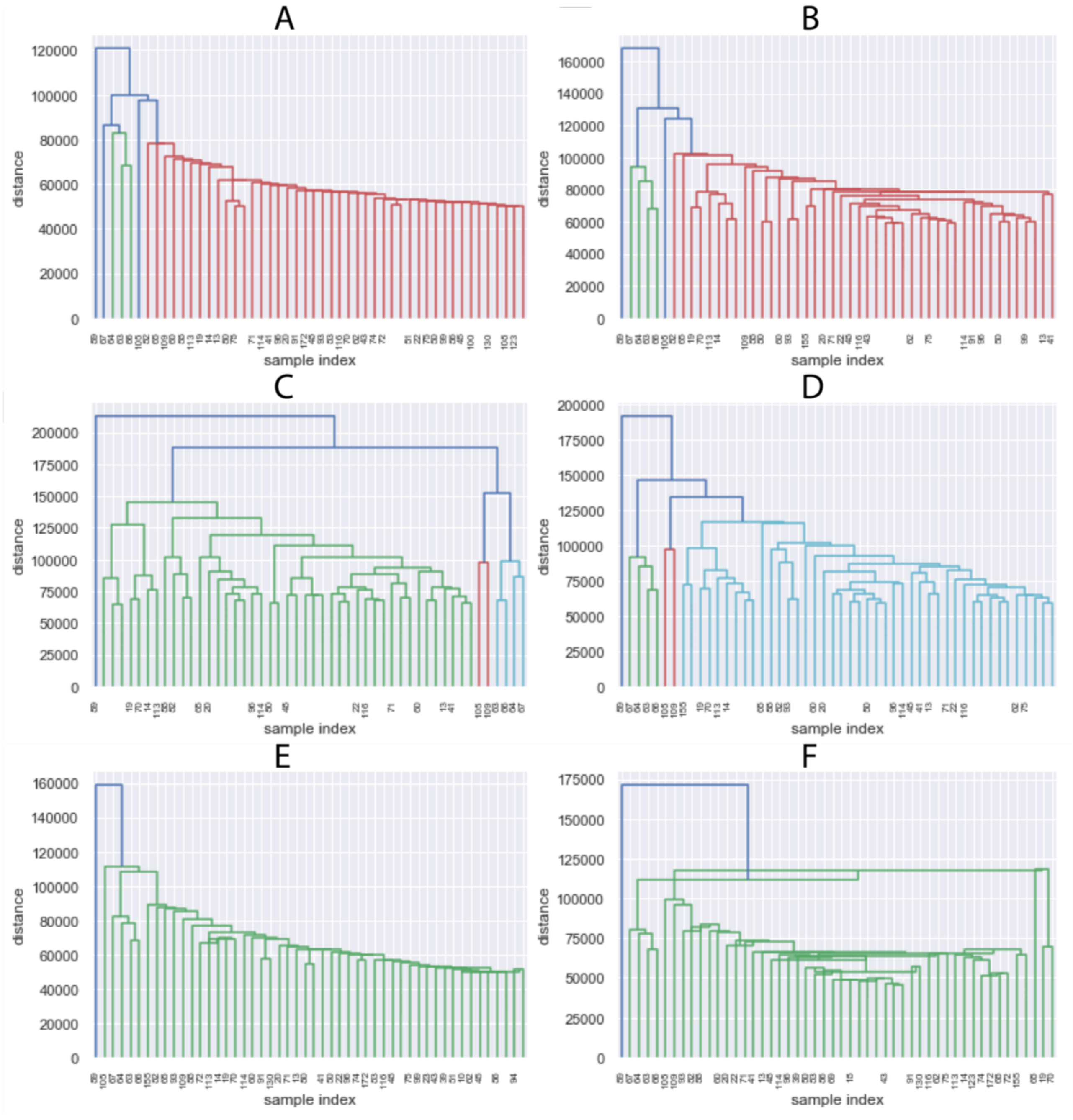

Hierarchical maps (dendograms) using as criterion for choosing the pair of clusters to merge at each step A) Single, B) Average, C) Complete, D) Weighted, E) Centroid, and F) Median methods. These hierarchical maps were generated by data without transformation and deleting their labels. Clustering approaches demonstrate whether the data contain relevant patterns for grouping.
